# 

**Published:** 1897-11-13

**Authors:** 


### EARLY EDITION.

**Notices of Births, Deaths, and Marriages** are inserted in this column at a charge of 2s. 6d. for an announcement not exceeding four lines.

### NOTICE TO CORRESPONDENTS.

All MS., Letters, Books for Review, and other matters intended for the Editor should be addressed The Editor, "The Hospital," The Hospital Building, 28 & 29, Southampton Street, Strand, W.C.

The Editor cannot undertake to return rejected MS., even when accompanied by stamped directed envelope.

**Notice to Advertisers and Subscribers.**

All Advertisements, Orders for Copies of The Hospital, and Business Communications should be addressed to **THE MANAGER, (Not to The Editor),** The Hospital, The Hospital Building, 28 & 29, Southampton Street, Strand, London, W.C.

The Cost of Subscription, post free, is as follows:—Per week, 2½d.; per quarter, 2s. 8d.; per half-year, 5s. 3d.; per annum, 10s. 6d. Foreign:—Per annum, 15s. 2d.; payable, in all cases, in advance.

### Scraps and Gleanings.

The street collections in Cork on Hospital Saturday amounted to about £230.

Mr. A. Walrond has given £100 towards the £2,000 required to clear the Devon and Exeter Hospital from debt.

The Victoria and Hatton wards at Addenbrooke's Hospital, Cambridge, are to be re floored with oak parquetry at a cost of about £280.

The Superioress of the North Charitable Infirmary, Cork, recently celebrated her jubilee of work as a sister of charity. She entered the nursing department of the infirmary in 1865.

Mr. Erasmus Kelly, secretary to the Nottingham General Hospital, has been presented with a handsome silver tea service as a wedding gift from those connected with the local Hospital Saturday Fund.

The committee of the Westbury Cottage Hospital have reported that in response to the appeals made, the building fund now amounts to about £460 and the maintenance fund to £78 in annual subscriptions.

The life of a night warder in a New Zealand hospital is apparently not a happy one if one may judge from the following incident. A patient, about three days after his admission, got out of bed, and on the night warder endeavouring to persuade him to return, knocked the warder down, and then proceeded to beat him about the head with a poker, inflicting severe wounds. Only the interference of another patient prevented a fatal result.

It is expected that the alterations necessary to convert the old leper hospital at Waterford into a county infirmary will be completed about January next. The alterations will cost about £8,000.

Quite recently a history of the Sheffield General Infirmary (now the Sheffield Royal Infirmary) from its establishment in 1789 to the end of the year 1895, has been published on behalf of the Infirmary Board.

The Children's Home, Much Hadham, Herts, have issued an explanation of the following item in their accounts:—"Christmas festival for children, £34 8s. 4d." This item, they explain, includes not only Christmas presents and rejoicings, but also a number of household things which have been long needed, such as new ward fenders, cot quilts, cripple's wheel chair, ward curtains, &c.

The constitution and rules for the government of the Glasgow Hospital Sunday Fund, prepared by the committee of management, have been approved by a general meeting of the contributors. The fund is to be under the control of a general committee, consisting of the chairmen of the House Committee of the Royal Infirmary and of the Boards of Management of the Western and Victoria Infirmaries, the Lord Provost of Glasgow, Sir James Bell, Bart., the promoter of the fund, and representatives from the contributing denominations or societies on the following scale: One representative for contributions of from £5 to £100; two representatives for contributions between £100 and £250; and one additional representative for every additional £250.

### The Student of Medicine.

### APPOINTMENTS.

Dr. C. Barker, appointed Medical Officer for the Haxey District of the Gainsborough Union. W. W. Chamberlain, M.B., C M.-Edin., appointed Medical Officer of Health for the Rawdon Urban District. C. M. Cooper, M.B., C.M., appointed House Surgeon to the Edinburgh Royal Maternity and Simpson Memorial Hospital. J. F. Counihan, L.R.C.P., L.R.C.S., L.M Irel., appointed Medical Officer to the Kilrush Workhouse. G. Dean, M.A., M.B., C.M., appointed Bacteriologist in the Antitoxin Department of the British Institute of Preventive Medicine. K. M. Douglas, M.D., F.R.C.S.Edin , appointed Medical Officer to the General Post Office, Edinburgh. Dr. Edwards, appointed Medical Officer for the Markyate District of the Hemel Hempstead Union. A. G. Goodwin, L.F.P.S.Glasg., L.M., appointed Medical Examiner to the Royal Liver Friendly Society at Liverpool. L. Grant, M.B., C.M , appointed House Surgeon to the Edinburgh Royal Maternity and Simpson Memorial Hospital. J. Guy, L.R.C.P., M.R.C.S.Eng., appointed Assistant Medical Officer of the Workhouse of the Bradford Union. W. Hughes, M.R.C.S.-Eng., appointed District Medical Officer of the Carnarvon Union. R. Moreton, M.R.C.S., L.R.C.P., appointed Visiting Surgeon to the Chester General Infirmary. G. H. Parry, M.B., C.M., appointed Special Non-Resident Clinical Clerk for the Observation Ward of the Edinburgh Royal Infirmary. T. Roberts, M B., C.M.Edin., appointed District Medical Officer of the Carnarvon Union. F. A. Spreat, M.R.C S Eng., D.P.H., appointed Medical Officer of Health to the Friern Barnet Urban District Council. C. L. H. Tripp, M.R.C.S Eng., appointed Medical Officer for the Dawlish District of the Newton Abbot Union. R. Wade, M.R.C.S.-L.S.A., appointed Medical Officer of Health to the Highbridge Urban District Council. D. C. Watson, M.B., C.M., appointed a Special Non-Resident Clinical Clerk for the Medical Waiting Room at the Edinburgh Royal Infirmary. G. E. Williams, L.S.A., appointed District Medical Officer of the Carnarvon Union. H. Witham, M.R.C,S., L.R.C.P.Lond., appointed Medical Officer for the Kirton District of the Boston Union.

